# Metformin Alleviates Doxorubicin-Induced Cardiotoxicity via Preserving Mitochondrial Dynamics Balance and Calcium Homeostasis

**DOI:** 10.1007/s12010-024-05141-9

**Published:** 2025-01-10

**Authors:** Nashwa Maghraby, Mona A. H. EL-Baz, Athar M. A. Hassan, Sary Kh. Abd- elghaffar, Amira S. Ahmed, Mahmoud S. Sabra

**Affiliations:** 1 Department of Medical Biochemistry, Faculty of Medicine, Assiut University, Assiut, 71515 Egypt; 2 Department of Medical Biochemistry, Badr University of Assiut, New Nasser City, Assiut, Egypt; 3 Department of Biochemistry, Faculty of Veterinary Medicine, Assiut University, Assiut, Egypt; 4 Department of Pathology and Clinical Pathology, Faculty of Veterinary Medicine, Assiut University, Assiut, Egypt; 5 Department of Pathology and Clinical Pathology, Faculty of Veterinary Medicine, Badr University of Assiut, New Nasser City, Assiut, Egypt; 6 Department of Histology and Cell Biology, Faculty of Medicine, Assiut University, Assiut, Egypt; 7 Department of Pharmacology, Faculty of Veterinary Medicine, Assiut University, Assiut, 71516 Egypt; 8 Department of Pharmacology, Faculty of Veterinary Medicine, Badr University of Assiut, New Nasser City, Assiut, Egypt

**Keywords:** Doxorubicin, Cardiotoxicity, Metformin, Mitochondrial dynamics, Calcineurin

## Abstract

Doxorubicin (DOX) is a commonly used chemotherapeutic medication for treating malignancies, although its cardiotoxicity limits its use. There is growing evidence that alteration of the mitochondrial fission/fusion dynamic processes accompanied by excessive reactive oxygen species (ROS) production and alteration of calcium Ca^2+^ homeostasis are potential underlying mechanisms of DOX-induced cardiotoxicity (DIC). Metformin (Met) is an AMP-activated protein kinase (AMPK) activator that has antioxidant properties and cardioprotective effects. The purpose of the study is to assess Met's possible cardioprotective benefits against DOX-induced cardiotoxicity. The study included 32 adult male rats. They were randomly divided into four groups: administered saline, DOX, Met, or DOX combined with Met respectively. Heart tissues were used for biochemical assays that measured oxidative stress markers, malondialdehyde (MDA), reduced glutathione (GSH), mitochondrial dynamics markers, optic atrophy-1(OPA-1) and dynamin-1-like protein (Drp1), calcineurin and caspase-3. Serum levels of myocardial injury markers, cardiac troponin I (cTn-I), and aspartate aminotransferase (AST), were also measured. The results revealed that DOX intoxication was associated with a significant increase in the levels of serum cTn-I and AST, increased cardiac MDA level, increased cardiac Drp1, calcineurin, and caspase-3 expressions, as well as reduced cardiac GSH level and cardiac OPA-1 expression. On the other hand, Met treatment significantly reduced DIC by decreasing oxidative stress, apoptosis, and improving mitochondrial and calcium balance. Finally, this study shows that Met may be able to protect the heart from damage caused by DOX by working as an antioxidant and anti-apoptotic agent and keeping the balance of calcium and mitochondria.

## Introduction

Anthracyclines are now a common component of many chemotherapy regimens. Both adults and children commonly use the prototypical anthracycline chemotherapeutic drug doxorubicin (DOX, trade name Adriamycin) to treat solid tumors and hematological malignancies [1]. Despite its effectiveness, up to 25% of individuals experience DOX-induced cardiotoxicity (DIC), restricting its clinical application [2].

The measured concentrations of doxorubicin and doxorubicinol in plasma were between 12.54–620.01 ng/mL and 1.10–27.00 ng/mL, respectively, while the measured cumulative dosages of doxorubicin ranged between 48.76–319.01 mg/m^2^, which is considered cardiotoxic in humans [3]. Following the administration of DOX, cumulative dose-dependent cardiotoxicity manifests as cardiomyopathy, which may ultimately lead to heart failure [4]. Clinical studies have indicated that individuals with cancer anthracycline-based chemotherapy may experience a reduction in heart mass, especially in the left ventricle. This decrease could serve as a biomarker for therapeutic interventions, as it is associated with increasing heart failure [5, 6]. One possible explanation for the decrease in cardiac mass after DOX treatment is the death of cardiac cells by apoptosis [7].

Although the pathogenesis of DIC is complicated and not completely understood [8]. Several mechanisms, such as intracellular production of excessive oxidative stress, loss of mitochondrial function, mitochondrial dynamic imbalance, and disturbance of Ca^2+^ homeostasis, can lead to DIC, all contributing to cardiomyocyte apoptosis and death [8, 9].

Mitochondria are highly active organelles that occupy around 40% of the volume of each cardiomyocyte, with the majority of the energy produced by the cardiomyocyte coming from mitochondrial respiration [10]. Mitochondrial dynamics is the term for the fission and fusion processes that occur continuously in mitochondria [11]. While mitochondrial fusion is driven by fusion proteins such as mitofusin 1 (Mfn1), mitofusin 2 (Mfn2), and optic atrophy-1 (OPA-1), fission protein 1 (Fis1) coordinates fission by recruiting the cytosolic GTPase dynamin-related protein 1 (Drp1) to cleave mitochondria [12].

Mitochondria are a major cellular target of DOX. DOX accumulation in cardiomyocyte mitochondria leads to increased reactive oxygen species (ROS) generation and reduced energy production, which in turn results in cell apoptosis. This excessive ROS accumulation disrupts mitochondrial dynamics and function, linking it to DIC [13–15].

Researchers have also demonstrated that DOX disrupts Ca^2+^ homeostasis. It can cause calcium overload in the mitochondria by triggering endoplasmic reticulum stress [9]. Numerous biological activities depend on calcium homeostasis. Calcium within cells regulates several important pathways, one of which is the calcineurin signaling cascade. Only prolonged Ca^2+^ elevation activates and expresses calcineurin, a Ca^2+/^calmodulin-dependent serine/threonine protein phosphatase. It regulates various. It regulates various processes, including cardiac apoptosis [16].

Based on all of these data, it appears that mitochondria play a significant role in the development of DIC, and maintaining mitochondrial homeostasis could be the best strategy to preserve cardiac cell function while undergoing anthracycline therapy [17]. Therefore, modulation of mitochondrial dynamic proteins using either fission inhibitors or fusion promoters can provide cardioprotection against DIC.

Metformin (Met) is an oral medication used as the primary treatment for type II diabetes [18, 19]. It has been determined to be a possible pharmaceutical intervention to lessen the cardiotoxicity caused by oncologic therapy. Previous investigations have revealed that Met exerted cardioprotection against DIC mostly through the decrease of oxidative damage, inflammation, and cardiac apoptosis [20, 21]. However, the effects of Met on mitochondrial dynamic balance and calcium homeostasis and their role in cardioprotection are still incompletely understood. Therefore, the current study aims to investigate the damaging mechanism of DOX on myocardium from the perspectives of mitochondrial dynamic imbalance and calcium dysregulation, as well as whether Met can alleviate DIC by restoring mitochondrial and calcium homeostasis.

## Materials and Methods

### Animals

The current study used 32 Wistar adult male albino rats weighing between 190 and 210 g each. The animals were obtained from the Faculty of Medicine's Animal House at Assiut University. They were permitted to acclimate for 7 days under regular laboratory circumstances (12-h light/dark cycle, standard temperature and humidity, and appropriate hygiene measures), with free access to food and clean drinking water. The Animal Research Ethics Committee, Faculty of Medicine, Assiut University, approved and provided guidance for the experimental protocols used (IRB local approval number 04–2023–300201).

### Experimental Design

After the period of acclimatization, animals were divided randomly into four groups (*n* = 8 each). The doses of DOX (3 mg/kg/IP) and metformin (250 mg/kg/p.o) were selected based on prior studies [20, 22–24]. **Control group:** rats injected intraperitoneally (IP) with normal saline (2.5 ml/kg) every other day for 14 days.** DOX group:** rats injected IP with DOX (3 mg/kg) every other day for 14 days for a total of six injections. The cumulative dose of DOX was 18 mg/kg. **Met group:** rats given Met (250 mg/kg/day) by oral gavage for 14 days. **DOX + Met group:** rats given both Met and DOX as described. Doxorubicin Hydrochloride (DOX·HCl) and metformin were purchased from Bio-Technology Co., Ltd. (Catalog no. J90044, Shanghai, China) and Pharma Quanao Chemical Co., Ltd. (Catalog no. RE977, China), respectively.

### Analysis of Blood Pressure (BP)

Before induction and weekly during the experiment, blood pressure (systolic and diastolic) was measured using a non-invasive tail-cuff technique (Model LE 5001-pressure meter, Panlab, Harvard Apparatus, Spain). To ensure that readings were accurate and in compliance with manufacturer specifications, rats were trained for three consecutive days prior to testing [25].

### Serum and Tissue Preparation

On day 15, before sacrifice, animals were accurately weighed and anesthetized using chloroform inhalation. Blood samples obtained from the retro-orbital venous plexus were centrifuged at 3000 rpm for 20 min in order to separate the serum. The serum was then stored in a deep freezer at −80 °C until utilized to measure the serum levels of myocardial injury markers; cardiac troponin I (cTnI) and aspartate aminotransferase (AST). Later, anesthetized animals were euthanized by decapitation. The hearts were removed and rapidly washed with ice-cold phosphate-buffered saline and weighed. The left ventricle (LV) of each heart was dissected and divided into 3 sections. The first portion was formalin-fixed and processed for histopathological and immunohistochemical analysis. The second part was frozen in liquid nitrogen and stored at −80 °C for further biochemical analysis. The third section was preserved in glutaraldehyde before being examined under an electron microscope.

### Measurement of Body Weight and Heart Weight

At the start and end of the experiment, all of the experimental animals' starting and final body weights (BWs) were measured and recorded. Following the animal scarification, the heart was removed, and the precise weight, as well as the relative heart weight (HW) to the BW, were determined. The differences between the test groups were observed.

### Histopathological and Immunohistochemical Analysis

The heart tissues of all tested groups were examined at histological and immunohistochemical levels. For the histopathological analysis, 5- to 7 μm thick sections of the heart's formalin-fixed paraffin-embedded tissue were used. The sections were then stained using standard hematoxylin and eosin (H&E) stains to examine the overall structure of the extracted tissue and Masson Trichrome stain to measure the amount of collagen using a light microscope [26].

For immunohistochemistry analysis, formalin-fixed, paraffin-embedded tissue slices were stained for caspase-3 polyclonal antibody (Catalog code: PA5-23,921 supplied by Thermo Scientific, USA) according to the manufacturer’s instructions. Immunohistochemical staining was performed using the streptavidin–biotin–peroxidase complex method [27].

### Immunohistochemical Analysis of Caspase-3 and Masson Trichrome Analysis of Collagen Fibers

The tissues were imaged using a high-resolution color digital camera attached to a microscope and connected to a computer. Image processing software (IMAGE-J, NIH OCI, and USA) was used to count the number of caspase-3 immunopositive cells as well as the percentage of collagen fibers in Masson Trichrome-stained sections. Measurements were taken in ten randomly selected non-overlapping fields at a magnification of 400 from the animals in each group [28, 29].

### Enzyme-Linked Immunosorbent Assay (ELISA)

The levels of cTnI and AST in the sera of different animals’ groups were measured using commercial ELISA kits (Catalog no. LS-F4164, supplied by LS Bio co. USA) and (Catalog no. MBS264975, supplied by MyBioSource, USA), respectively, according to the manufacturer's specifications.

### Colorimetric Assay

Frozen specimens of left ventricles were thawed, followed by homogenization in 5–10 ml cold buffer (50 mM potassium phosphate, pH 7.5) per gram tissue using tissue Glass-Col Homogenizer. The homogenates were centrifuged at 4,000 rpm for 15 min at 4 °C. The supernatant was removed, and the levels of malondialdehyde (MDA) and reduced glutathione GSH were assayed spectrophotometrically using commercial colorimetric kits (Catalog no.GR 2511, Biodiagnostics, Egypt) and (Catalog no. MD 25 29, Biodignostics, Egypt), respectively, according to the manufacturer's specifications [30–32].

### RNA Extraction and Quantitative Real-Time PCR (qPCR)

The total RNA was extracted from the frozen, gathered heart tissues of the investigational animals with the aid of the RNeasy Mini Kit (Catalog no. 74104, Qiagen, Germany) according to the protocol of the manufacturer. Following quantitation using a nanodrop spectrophotometer (Epoch Microplate Spectrophotometer, Biotech, VA, USA), the extracted RNA (500 ng) was used to construct the complementary DNA (cDNA) with the High-Capacity cDNA Reverse Transcription kit with RNase inhibitor (Catalog no. 4374966, Thermo-Fischer Scientific, USA). Then, cDNA was amplified using the Maxima SYBR Green qPCR Master Mix kit, (Catalog no. #K0251, Thermo-Fischer Scientific, USA) and used as a template for the following target genes: OPA-1, Drp1, and calcineurin. The amplification was performed using the primer sets described in Table 1. Specific conditions of RT-qPCR were as follows: an initial denaturation cycle of 95 °C for 10 min, followed by 40 amplification cycles of 95 °C for 15 s and 60 °C for 1 min using the Applied Biosystems 7500 Fast Real-time PCR machine (Applied Biosystems, Germany). The data was normalized against the control β-actin and shown as relative mRNA expression (fold change) using the 2 − ΔΔCT technique [33].

**Table 1:** The sequences of the PCR primers

Gene	5′−3′ primer sequence
Drp-1	**Forward:** AGTAAGCCCTGAGCCAATC **Reverse:** GGGATTACTGATGAACCGAAG
OPA	**Forward:** CAGCTGGCAGAAGATCTCAAG **Reverse:** CATGAGCAGGATTTTGACACC
Calcinurein	**Forward:** CTGAGATGCTGGTAAACGTCCTGA **Reverse:** TGCTCGGATCTTGTTCCTGATG
β- actin	**Forward:** CACTATCGGCAATGAGCGGTTCC **Reverse:** CAGCACTGTGTTGGCATAGAGGTC

### Electron Microscopy Evaluation

For electron microscopy examination: left ventricle specimens were fixed in 5% glutaraldehyde for at least 24 h. Semi-thin slices (0.5–1 μm) were then prepared and stained with toluidine blue before being inspected under a light microscope and photographed. Select areas of semi-thin sections were sliced into ultra-thin sections (500–800 nm), which were then collected on copper grids and contrasted with lead citrate and uranyl acetate. They were examined and photographed by the transmission electron microscope JEOL (JEM-100 CX11, TOKYO, JAPAN) at 80 kV in Assiut University's Electron Microscope Unit [23].

### Statistical Analysis

The statistical analyses were carried out using GraphPad Prism software (Version 5). A one-way analysis of variance (ANOVA) was employed to assess differences between the experimental groups, followed by a Tukey post hoc test. For repeated measures (blood pressure), data were analyzed using two-way ANOVA with repeated measures, followed by Tukey's multiple comparisons test. Data are presented as mean ± SD. *P* < 0.05 indicates statistical significance.

## Results

### Metformin's Impact on Arterial Blood Pressure

**Fig. 1:**
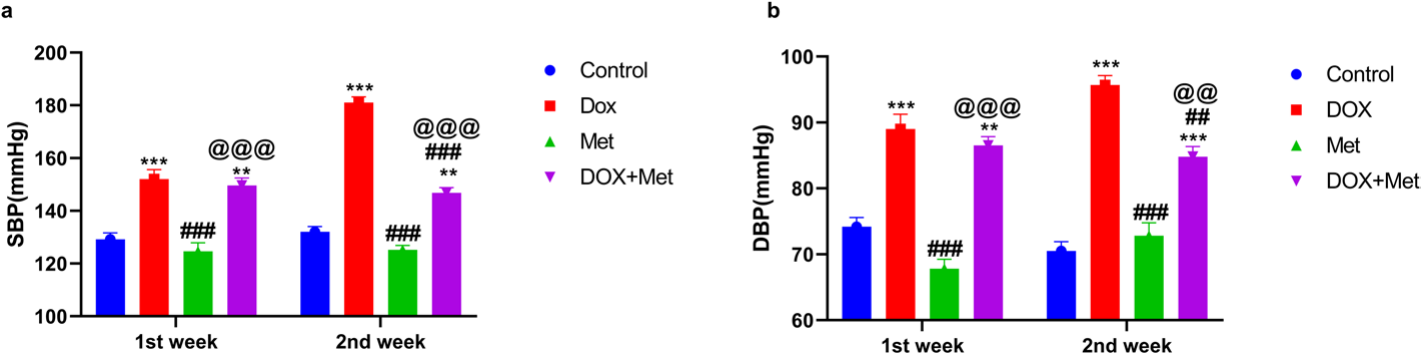
The impact of metformin (Met) on the systolic (**a**) and diastolic (**b**) blood pressure in the DOX-intoxicated rats. *n* = 8 per group. The data represents the means ± SD. ^**^p ≤ 0.01, ^***^p ≤ 0.001 vs. control group; ^##^*p* ≤ 0.01, ^##^*p* ≤ 0.001 vs. DOX-treated group; ^@@^*p* ≤ 0.01, ^@@@^*p* ≤ 0.001 vs. DOX: doxorubicin group; Met: metformin group; DOX + Met: doxorubicin + metformin group

The reference range for arterial blood pressure in the control group of rats was 124.6 ± 3.3 mmHg for systolic blood pressure (SBP) and 74.17 ± 1.4 mmHg for diastolic blood pressure (DBP). The results demonstrated that, in comparison to the control group, DOX treatment significantly raised the SBP and DBP in rats (Fig. 1). SBP increased from 129.2 ± 2.417 mmHg to 152 ± 3.592 mmHg in the first week and to 181 ± 2.2 mmHg in the second week after treatment. DBP levels also increased in the DOX group, reaching 89 ± 2.27 mmHg in the first week and 95.67 ± 1.43 mmHg in the second week. Co-administration of Met with DOX significantly diminished the elevation in blood pressure induced by DOX, resulting in a SBP of 146.8 ± 2 mmHg and a DBP of 84.83 ± 1.52 mmHg.

### Effect of Metformin on Body Weight and Heart Weight

Statistical analysis showed that the initial (BW) was similar among all of the groups (*p* > 0.05). However, treatment with DOX significantly (*P* < 0.001) decreased BW in DOX group compared to control group (180.9 ± 6.36 vs. 218.75 ± 3.77 g). Treatment with Met 250 mg/kg in DOX-intoxicated animals prevented BW reduction (*p* < 0.01) when compared to the DOX group (206.8 ± 5.39 vs. 180.9 ± 6.36 g). Furthermore, the DOX group's heart weight (HW) was significantly lower (*P* < 0.001) than that of the control group and animals that received Met only (0.67 ± 0.77 vs. 0.94 ± 0.08 g). Treatment with Met in DOX-intoxicated animals was found to prevent HW reduction compared with DOX groups (0.82 ± 0.06 vs. 0.67 ± 0.77 g). Our results also revealed a decrease in the HW to BW (HW/BW) ratio in the DOX-treated animals compared to the control group (Table 2).

**Table 2:** Body weight, heart weight and heart weight / body weight ratios of different groups

Variables	Control	DOX	Met	DOX + Met
Initial BW(g)	198.63 ± 5.24	198.38 ± 6.23	198.4 ± 5.45	199.8 ± 4.95
Final BW (g)	218.75 ± 3.77	180.9 ± 6.36 a***	217.3 ± 6.84 b***	206.8 ± 5.39 a**b**c**
HW (g)	0.94 ± 0.08	0.67 ± 0.77 a***	0.93 ± 0.09 b***	0.82 ± 0.06 a*b**c*
HW/BW (ratio%)	0.43 ± 0.03	0.37 ± 0.04 a**	0.43 ± 0.03 b*	0.4 ± 0.03

DOX: doxorubicin group; Met: metformin group; DOX + Met: doxorubicin + metformin group

Data are expressed as mean ± SD (*n* = 8 in each group) and analyzed by One-way analysis of variance (ANOVA) followed by Tukey test

a Significantly different from the value in the control group

b Significantly different from the value in the DOX-treated group

c Significantly different from the value in the Met-treated group

^***^p ≤ 0.001, **p ≤ 0.01, and *p ≤ 0.05

### Histopathological Results

**Fig. 2:**
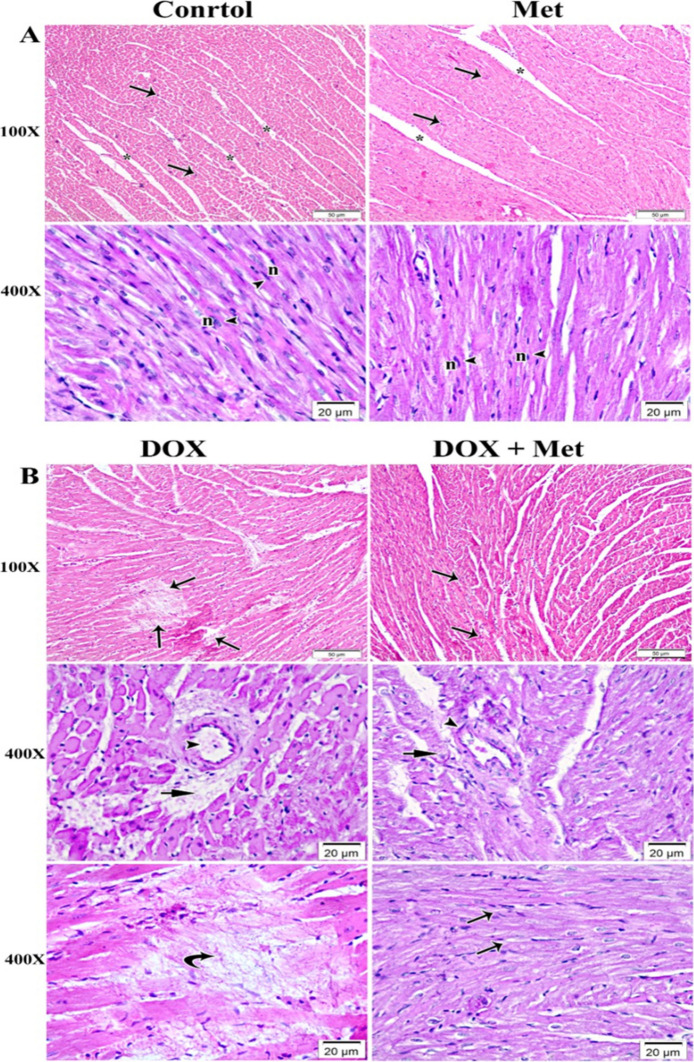
Photomicrograph of myocardium of left ventricle stained with hematoxylin and eosin (H&E). *n* = 8 per group. **A** The myocardium of the control and Met groups, showed normal architectural features of cylindrical irregular branched muscle fibers (arrows) separated by slit-like narrow spaces of endomysium (*). The branched myofibers contained central oval vesicular nuclei (n) and acidophilic sarcoplasm (arrow heads). **B** The myocardium of DOX group showed marked deterioration within the myocardial fibers (arrows). With high magnification, endothelial cell injury of the blood vessels (arrow heads) and perivascular hemorrhage (short arrows) were associated with deteriorated myofibers. Replacement of necrotic myocardial muscles with activation of a fibrocystic cell reaction was also revealed (curved arrows). In the group that received DOX and Met, the myocardium showed a small area of cellular deterioration (arrow heads) and mild vascular wall degeneration (short arrow) that was associated with many normally arranged myocardial fibers (arrows). DOX: doxorubicin group; Met: metformin group; DOX + Met: doxorubicin + metformin group

The findings revealed that H&E heart sections from rats given Met were nearly identical to those of the control group. The control group's analysis revealed a classical histological pattern of cardiac muscle fibers. The cardiac muscle fibers were running in different directions and exhibited pale eosinophilic sarcoplasm. They had nuclei that were oval, vesicular, and centrally located, with minimal striations. The interstitial spaces were detected as small slit-like gaps between branching and anastomosing fibers (Fig. 2A). On the other hand, the heart sections of DOX-intoxicated rats showed histological alterations in muscle fibers, with areas of degeneration and interrupted muscle fibers, wide interstitial spaces, and perivascular hemorrhage associated with myocardial necrosis (Fig. 2B). Regarding the cardiac sections of the DOX group given Met treatment, they revealed a small area of cellular and vascular degeneration that is associated with many normally arranged myocardial fibers (Fig. 2B).

**Fig. 3:**
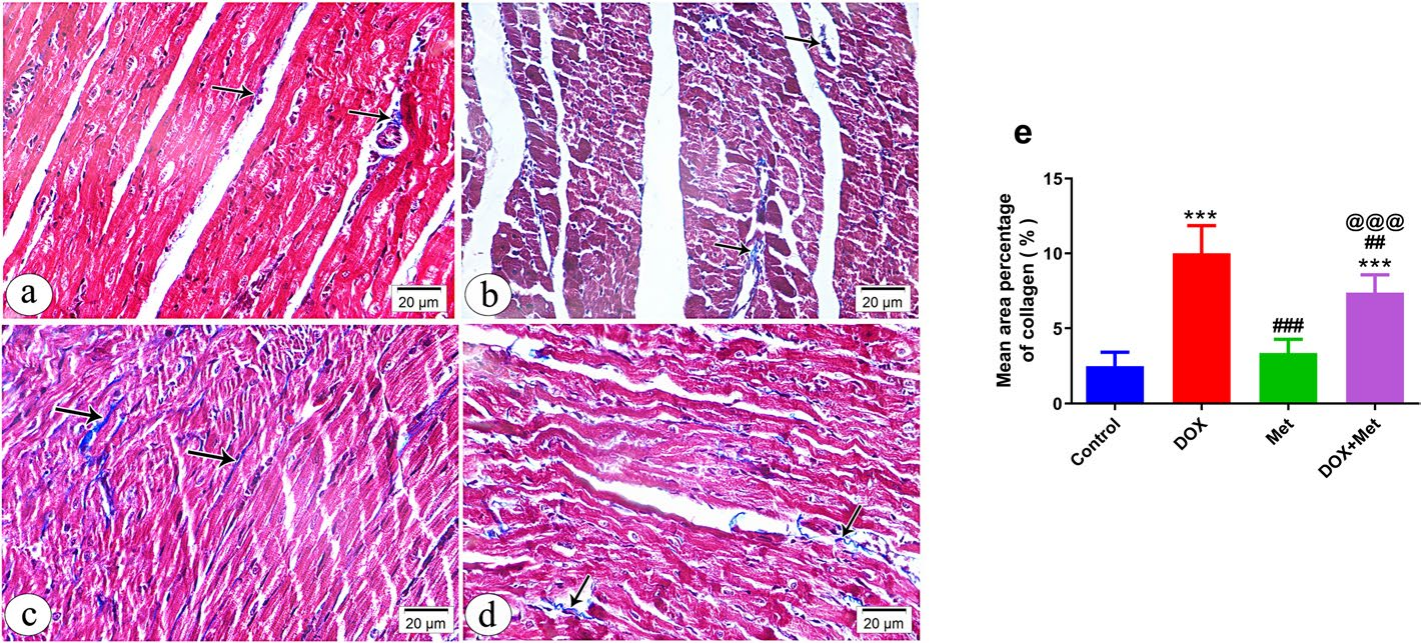
A photomicrograph of myocardium of left ventricle of different groups stained with Masson Trichrome stain at 400 × magnification (**a-d**). *n* = 8 per group. **a** Control group showing fine collagen fibers (arrows) between the cardiac muscle fibers and around blood vessels (arrowhead). **b** Group received DOX only showing increased amount of collagen fibers (arrows) between the muscle fibers compared to the control. **c** Group received Met only exhibiting minimal amount of collagen fibers (arrows) between the cardiac muscle fibers. **d** Group received DOX and Met showing moderate amount of collagen fibers (arrows) between the muscle fibers. **e** Histogram showing the quantitative estimation of the mean area percentage of collagen fibers in Masson Trichrome-stained sections. *n* = 8 per group. Data are expressed as mean ± SD. ^***^p ≤ 0.001 vs. control; ^##^*p* ≤ 0.01, ^###^*p* ≤ 0.001 vs. the DOX-treated group; ^@@@^*p* ≤ 0.001 vs. Met-treated group. DOX: doxorubicin group; Met: metformin group; DOX + Met: doxorubicin + metformin group

In addition to H&E examination, we examined collagen deposits in heart specimens stained with Masson Trichrome stain in different animal groups (Fig. 3a-e). The control group's myocardium revealed tiny collagen fibers scattered between muscle fibers and surrounding blood vessels (Fig. 3a), while the DOX-intoxicated group showed more collagen fibers between muscle fibers than the control group (Fig. 3b). The Met-treated group displayed a reduced amount of collagen fibers, almost identical to the control group (Fig. 3c). However, the DOX-intoxicated rats treated with Met had a moderate accumulation of collagen fibers between muscle fibers (Fig. 3d). A significant increase in the area percentage of collagen was observed in heart sections of DOX-intoxicated rats, which was reversed by Met treatment, indicating that Met treatment could have the potential to reduce collagen deposition in the heart (Fig. 3e).

### Effect of Metformin on Myocardial Injury and Oxidative Stress in the DOX- Intoxicated rats

**Fig. 4:**
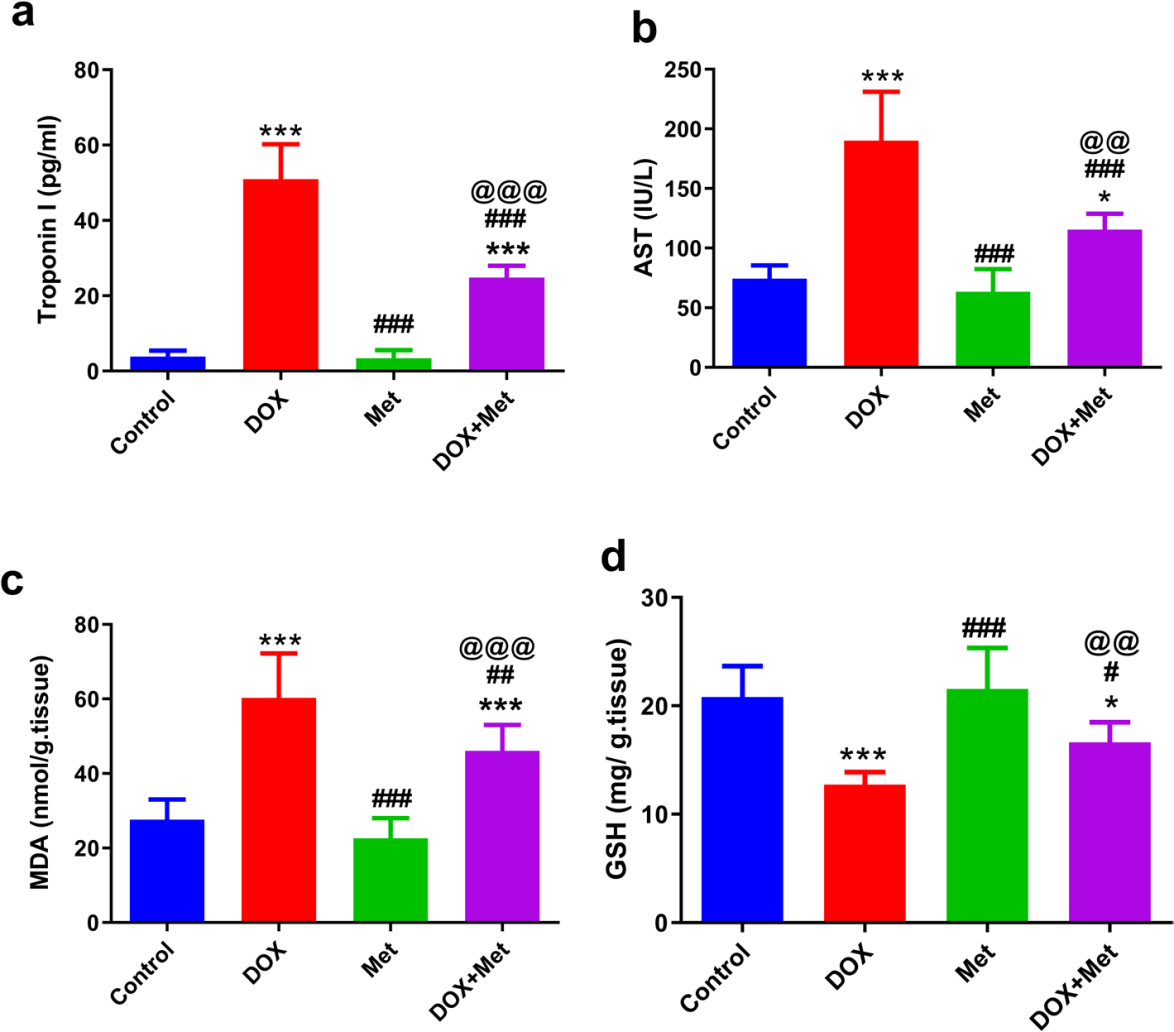
The effects of metformin treatment on cardiac injury and oxidative stress in the DOX-intoxicated rats. *n* = 8 per group. **a** Serum cTn-I levels, **b** Serum AST levels, **c** Tissue MDA levels, **d** Tissue GSH levels. *n* = 8 per group. Data are expressed as mean ± SD. ^*^p ≤ 0.05, ^***^p ≤ 0.001 vs. control group; ^#^*p* ≤ 0.05, ^##^*p* ≤ 0.01, ^###^*p* ≤ 0.001 vs. DOX-treated group; ^@@^*p* ≤ 0.01, ^@@@^*p* ≤ 0.001 vs. Met-treated group. AST: Aspartate aminotransferase; cTn-I: cardiac troponin-I; DOX: doxorubicin; DOX + Met: doxorubicin + metformin group, GSH: glutathione; MDA: malondialdehyde; Met: metformin

The extent of DIC damage was assessed by measuring serum cTn-I and AST levels **(**Fig. 4a and b). DOX administration was found to significantly increase (*p* < 0.001) serum levels of cTnI and AST compared with their levels in the sera of the control group (50. 94 ± 9.3 vs. 3.85 ± 1.59 pg/ml) and (190.1 ± 41.07 vs. 74.50 ± 11.10 IU/L), respectively. However, as compared to DOX-intoxicated rats, treatment with Met 250 mg/kg significantly (*p* < 0.001) decreased serum levels of cTn-I and AST (24.81 ± 3.18 vs. 50.94 ± 9.3 pg/ml) and (115.5 ± 13.32 vs. 190.1 ± 41.07 IU/L), respectively. These results suggest that Met could mitigate DIC.

In addition, cardiac tissues were used to determine the levels of the oxidative stress markers (MDA) and the antioxidant molecule, GSH, in different animal groups. As shown in Fig. 4c and d, DOX administration resulted in a significant (*P* < 0.001**)** increase in cardiac MDA (60.32 ± 11.9 vs 27.62 ± 5.45 nmol/g tissue); however, cardiac GSH was significantly (*P* < 0.001**)** decreased (12.74 ± 1.15 vs. 20.80 ± 2.86 mg/g tissue) in DOX-intoxicated rats in comparison to their levels in the cardiac tissues of the control group. Met significantly mitigated these effects of DOX. Met treatment induced a significant decrease (*P* < 0.01) in cardiac MDA (46.11 ± 6.91 vs. 60.32 ± 11.9 nmol/g tissue) as well as a significant increase **(***P* < 0.05**)** in cardiac GSH (16.63 ± 1.86 vs 12.74 ± 1.15 mg/g tissue) of intoxicated rats in comparison to the DOX-intoxicated group.

### Effect of Metformin on Mitochondrial Dynamics in the DOX- Intoxicated Rats

**Fig. 5:**
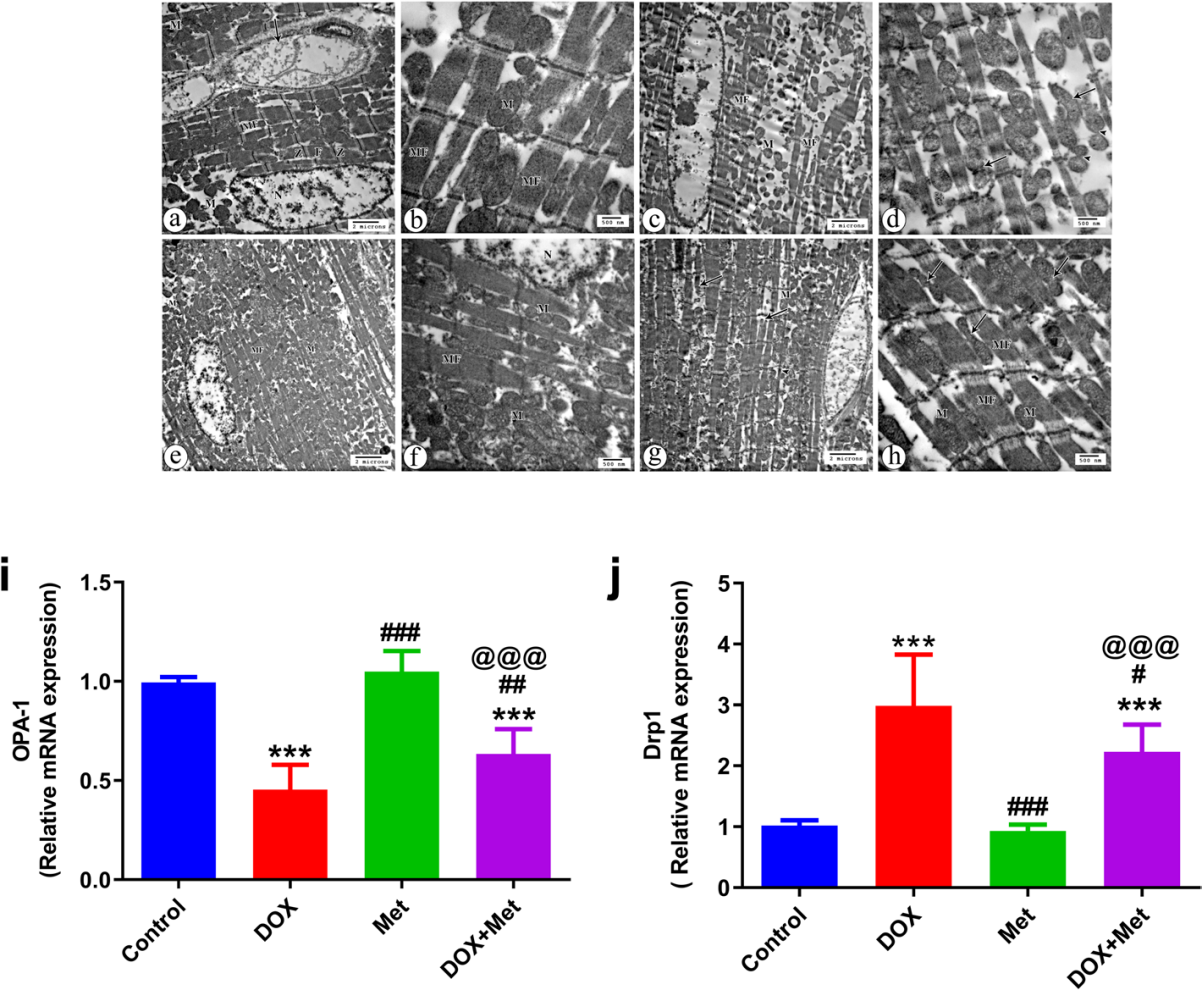
The effects of metformin treatment on cardiac mitochondrial dynamics in the DOX-treated rats. **a-h** Analysis of mitochondrial dynamics using transmission electron microscopy (TEM). The control group shows large euchromatic nucleus (N), parallel arrangement of myofibers (MF). In addition, the mitochondria (M) are observed arranged perinuclear and in rows between the myofibers with regularly arranged sarcomeres between Z lines (Z), regular arrangement of the myofibrils (F) within the sarcomeres between Z lines (Z), and endomysium blood capillaries (arrow) (X 4800) (**a**). Distribution of mitochondria (M) in between well-organized myofibers (F) (X 14000) is also noticed (**b**). The group that received Dox only shows a prominent myofibrillar loss and disarray (MF) with distribution of spaced mitochondria (M) in between disorganized and destructed myofibers (X 4800) (**c**). Marked abnormalities in mitochondrial morphology that include contour irregularities with fission (arrows) and fusion (arrow heads) in shape are also noticed (X 14000) (**d**). Group received Met only, shows equal or more apparent pathological changes in myofibers arrangement (MF) with mitochondria (M) distributed perinuclear and in rows between the myofibers (X 4800) (**e**). The group received DOX and Met, shows the distribution of mitochondria (M) between myofibers (MF) with relatively normal configuration and part of euchromatic nucleus (N) (X 14000) (**f**). Some pathological changes with disorganization and destruction of few myofibers (arrows) and thinning of others (arrowhead) are present. Notice the distribution of the mitochondria (M) in between the myofibers (X 4800) (**g**). More or less parallel arrangement of myofibers (MF) and distribution of mitochondria (M) in rows in between the myofibers, and some mitochondria with fission morphology are observed (arrows) (X 14000) (**h**). **i** and **j** Relative gene expression of mitochondrial dynamics markers, OPA-1 and Drp1. β actin was utilized to normalize expression data. *n* = 8 per group. Data are expressed as mean ± SD. ^***^p ≤ 0.001 vs. control group; #p ≤ 0.05, ^##^*p* ≤ 0.01, ^###^*p* ≤ 0.001 vs. DOX-treated group; ^@@@^*p* ≤ 0.001 vs. Met-treated group. DOX: doxorubicin; DOX + Met: doxorubicin + metformin group; Drp1: dynamin-related protein 1; Met: metformin; OPA-1: optic atrophy type 1

To explore the underlying molecular mechanisms involved in the action of metformin in mitochondrial protection against DIC, we examine mitochondrial dynamics using transmission electron microscopy (TEM). As shown in (Fig. 5a–h), the control group showed regular arrangement of the myofibrils within the sarcomeres and regularly arranged sarcomeres between Z lines. The myocardial fiber contained a large oval euchromatic nucleus and mitochondria, which were arranged perinuclearly and in rows between the parallel arrangement of myofibers (Fig. 5a and b). Group treated with Met only showed equal or more apparent pathological changes in myofibers with regular arrangement of the myofibrils within the sarcomeres between Z lines. Mitochondria also distributed perinuclear and in rows between parallel arranged myofibers with normal configuration (Fig. 5e and f). On the other hand, cardiac tissues of the DOX- treated group destructed myofibers with a prominent myofibrillar loss and disarray in addition to spaced mitochondria in between the disorganized myofibers (Fig. 5c). Mitochondrial morphology also showed marked abnormalities in the shape that included contour irregularities with fission and fusion (Fig. 5d). In the DOX group treated with Met, the cardiac tissues showed some pathological changes in the form of disorganization and destruction of a few myofibers and thinning of others (Fig. 5g). Arrangement of variable mitochondria was observed in between more or less organized myofibers, some of them with fission morphology (Fig. 5h).

In addition to TEM analysis, we measured the gene expression of mitochondrial dynamics markers, OPA-1 and Drp1. The result revealed that, DOX treatment promoted mitochondrial fragmentation by stimulating the expression of Drp1, the mitochondrial fission marker, and inhibiting the expression of OPA-1, the mitochondrial fusion marker. Drp1 expression was significantly upregulated (*P* < 0.001), however OPA-1 expression was significantly downregulated (*P* < 0.001) in the cardiac tissues of the DOX treated groups compared to those in control groups. The expression of Drp1 and OPA-1 were partially reversed by Met treatment. Drp1 was significantly downregulated (*P* < 0.05), meanwhile OPA-1 was significantly upregulated (*P* < 0.01) in the cardiac tissues of DOX group treated with Met compared to their expressions in the heart tissues of DOX-only treated group (Fig. 5i and j).

### Effect of Metformin on Calcium Homeostasis and Apoptosis in the DOX- Intoxicated Rats

**Fig. 6:**
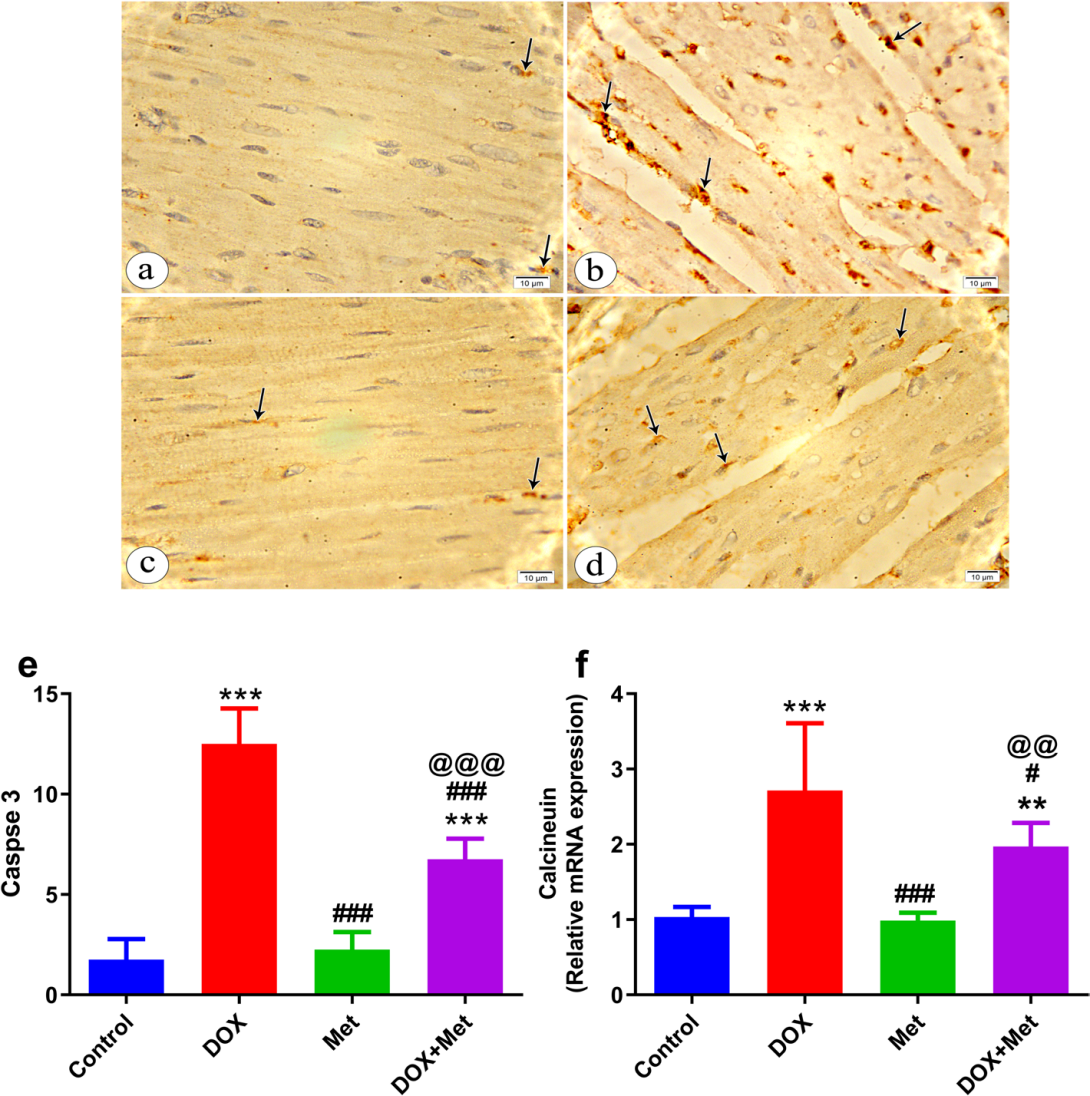
The effects of metformin treatment on cardiac calcium homeostasis and apoptosis in DOX-intoxicated rats. **a-e** A photomicrograph of myocardium of left ventricle immunohistochemically stained with caspase 3 (× 1000). Control group shows faint positive immunostaining reaction within the sarcoplasm of minimal myocardial cells (arrows) (**a**). Group received DOX only shows marked positive immunostaining reaction within the sarcoplasm of many myocardial cells (arrows) (**b**). Group received Met only treated group shows mild positive immunostaining reaction within the sarcoplasm of few myocardial cells (arrows) (**c**). Group received DOX and Met showing moderate positive immunostaining reaction in the sarcoplasm of some myocardial cells (arrows) (**d**). Histogram showing the quantitative estimation of the mean count of caspase-3 positive cells (**e**). **f** Relative gene expression of calcineurin. β actin was utilized to normalize expression data. *n* = 8 per group. Data are expressed as mean ± SD. ^**^p ≤ 0.01, ^***^p ≤ 0.001 vs. control group; ^#^*p* ≤ 0.05, ^###^*p* ≤ 0.001 vs. DOX-treated group; ^@@^*p* ≤ 0.01, ^@@@^*p* ≤ 0.001 vs. Met-treated group. DOX: doxorubicin; Met: metformin; DOX + Met: doxorubicin + metformin group

To investigate the effect of Met on calcium dysregulation, we compared the relative mRNA expression of the calcineurin in different animal groups. The results revealed that DOX treatment significantly increased relative mRNA expression of calcineurin (*P* < 0.001) in cardiac tissues compared to the control group. Interestingly, the Met treatment reversed the increase in calcineurin gene expression manifested by significant downregulation (*P* < 0.01**)** in calcineurin relative mRNA in the heart tissues of the DOX group treated with Met compared to the DOX-only treated group (Fig. 6f).

The impact of Met on DOX-induced cardiac apoptosis was also investigated. Immunohistochemical examination of cardiac tissue specimens further examined the influence of metformin treatment on DOX-mediated caspase 3 expression (Fig. 6a-e). As shown in (Fig. 6e) myocardial apoptosis was significantly increased in DOX-intoxicated rats as shown by a significant increase (*P* < 0.001**)** in caspase-3 expression with intense immune reactivity brown-colored staining (12.5 ± 1.77 Vs. 1.75 ± 0.1.044) compared to the control. On the other hand, Met significantly decreased (*P* < 0.001) caspase-3 expression in comparison to rats in the DOX-intoxicated group (6.75 ± 1.04 vs. 12.5 ± 1.77).

## Discussion

Doxorubicin is a potent anticancer medication. It is crucial in treating several cancerous conditions but can cause ventricular dysfunction and cardiomyopathy, a fatal illness that results in congestive heart failure with a fatality rate of around 50% [34, 35].

There is cumulative evidence that various molecular mechanisms are involved in DIC. They include mechanisms dependent on mitochondrial dysfunction such as DOX's impact on mitochondrial dynamic balance, redox cycling, oxidative stress, calcium dysregulation, and apoptotic pathways [36, 37].

Metformin is an oral antihyperglycemic drug commonly used to treat type II diabetes. Research has suggested that it possesses antioxidant properties and cardioprotective effects [38, 39]. Therefore, the current study was carried out to examine Met's ability to prevent DIC as well as its possible molecular mechanisms via measuring oxidative stress markers (MDA, GSH), mitochondrial dynamics parameters, calcineurin, and serum myocardial injury markers (cTn I and AST), along with a histopathological, immunohistochemical, and EM analysis of the heart tissue.

DOX has been shown to increase myocardial injury markers, which are a consequence of the multiple mechanisms involved in DIC [40]. Previous studies reported that Met reduced the increased levels of cardiac injury biomarkers cTn I and AST compared to DOX-intoxicated rats [8, 22, 41]. In agreement, our data indicated that DOX increased both serum cTn I and AST levels while Met treatment could reduce their levels, indicating the potential protective effect of Met on the cellular architecture of the myocardium against DIC.

Many studies indicated that oxidative stress is the primary mechanism for DIC [42–44]. ROS are produced as DOX is oxidized to semiquinone, an unstable metabolite that is later turned back into DOX [45]. This agent generates an excess of ROS in the mitochondria, causing oxidative damage to biological macromolecules such as lipids, proteins, and DNA as well as altering the structure and function of cardiac cell membranes [46]. Because cardiomyocytes have fewer defenses from antioxidant enzymes, DOX lowers endogenous antioxidants and increases lipid peroxidation, compromising cardiac function [47].

Previous studies demonstrated that DOX promoted excessive lipid peroxidation, as indicated by an elevated MDA level and decreased cardiac levels of GSH [8, 48–50]. Interestingly, Met has been reported to effectively reduce oxidative damage by decreasing MDA levels and increasing the levels of antioxidants, including GSH, in the DOX-treated rats [8, 23]. Consistently, our study revealed that DOX intoxicated rats experienced higher levels of MDA and decreased GSH levels in the cardiac tissues. On the other hand, Met treatment was found to attenuate oxidative stress, as indicated by increased cardiac GSH with decreased MDA cardiac levels in the DOX-treated rats.

The present investigation demonstrated that a 14-day therapy with DOX resulted in a reduction in BW, HW, and the HW/BW ratio. Likewise, previous study indicated marked reduction in the values of BW, HW, and the HW/BW ratio in DOX treated rats in comparison to the other groups. The researchers ascribed these findings to diminished hunger in DOX-treated rats and the suppression of protein synthesis induced by DOX [22]. Additional research indicated no substantial difference among the examined groups [51, 52]. Nonetheless, prior research indicated that a 14-day therapy with DOX resulted in an elevation of heart weight and the HW/BW ratio. The augmentation of heart weight may be ascribed to the enlarged, dilated, and hypertrophied atria and ventricles [53]. In our investigation, Met therapy in DOX-intoxicated rats was observed to avert HW decline relative to the DOX groups.

Mitochondria account for approximately 40% of cardiac volume and play a critical role in regulating ROS generation, energy metabolism, calcium homeostasis, and cell death signaling in cardiomyocytes [10, 54, 55]. The intracellular organelles with the most damage from DOX exposure are the mitochondria, and it is thought that mitochondrial damage is one of the initial events of cardiotoxicity [56]. One major mechanism related to DOX-induced cardiac mitochondrial damage is the impaired function of mitochondrial dynamics proteins, which regulate mitochondrial fusion and fission [17, 57, 58]. An increase in mitochondrial fragmentation following mitochondrial fission events is frequently associated with mitochondrial dysfunction, with this aberrant morphology predominating under high stress levels and cardiac cell death [59, 60].

Previous studies demonstrated that DOX suppressed mitochondrial fusion processes and induced excessive mitochondrial fission in cardiomyocytes [61, 62]. Interestingly, a recent study found that Met could support the balance of cardiac mitochondrial dynamics by increasing mitochondrial fusion proteins (Mfn-1, Mfn-2, and OPA-1) and decreasing mitochondrial fission protein (Drp1) in DOX-treated rats [8].

In line with previous studies, our results revealed that DOX treatment promoted mitochondrial fragmentation by inducing the expression of Drp1 and inhibiting the expression of mitochondrial fusion protein OPA-1. On the other hand, treatment with Met effectively attenuated the deleterious effects of DOX on cardiac mitochondria and promoted the balance of cardiac mitochondrial dynamics via increasing in OPA-1 expression and decreasing Drp1 expression in the cardiac tissue of DOX-intoxicated rats. These results were also confirmed by EM analysis, which revealed the DOX group treated with Met were almost quite similar to the control with some fission in morphology.

Apart from mitochondrial damage, calcium dysregulation is another well-known and established mechanism that contributes to DIC [36]. Several investigations have demonstrated that administration of DOX results in elevated Ca^+2^ levels and ROS production in various types of cells, hence activating both intrinsic and extrinsic cell death [63–66]. Calcium homeostasis is known to be impacted by doxorubicinol, the hydroxyl metabolite of DOX, by many mechanisms. These include the modification of the sodium/potassium exchanger on the sarcolemma and the sarco/endoplasmic reticulum Ca21 ATPase (SERCA) found on the sarcoplasmic reticulum (SR) [67, 68]. Moreover, increased mitochondrial fission and inhibited fusion in cardiomyocytes due to DOX intoxication has been reported to result in mitochondrial morphological abnormality and dysfunction, accompanied by excessive ROS production, intracellular Ca^2+^ overload, and Ca^2+^ signaling, contributing to events leading to cell death and cardiac dysfunction [66, 69, 70].

Calcineurin, a Ca^2+^calmodulin-dependent serine/threonine protein phosphatase that is only produced during prolonged Ca^2+^ elevation and is involved in the control of numerous processes, including cardiac apoptosis, can be activated by prolonged increased intracellular calcium levels [16]. Increased calcium levels lead to a calcineurin-dependent activation of the nuclear factor of activated T-lymphocytes, which further enhances the Fas mediated cardiac cell death [66]. Activated calcineurin also dephosphorylates Drp1 at Ser637, thus mediating Drp1 translocation from the cytosol to the mitochondria for activation of the fission process [71].

Previous studies indicated increased calcineurin protein expression and activity as well as caspase −3 expression on DOX treatment [66, 72, 73]. Consistently, previous clinical studies showed that DOX causes an early reduction in cardiac mass in cancer patients as a result of cardiomyocyte death [5, 6]. In agreement with the above-mentioned outcomes, our results showed increased calcineurin gene expression in DOX-intoxicated rats. In addition, immunohistochemical analysis of rat heart tissue revealed increased caspase −3 expression upon DOX treatment. These findings were confirmed by substantial reduction of heart weight in DOX treated rats compared to control. Notably, it has been reported that caspase inhibition during early reperfusion could safeguard the myocardium from fatal reperfusion injury [74, 75].

Regarding the effect of Met on calcineurin and caspase-3 expressions, our results showed that Met treatment significantly prevented the elevation of calcineurin and caspase-3 expressions and thus cardiac apoptosis. In concomitant with our results, previous study demonstrated that administration of Met to DOX-intoxicated rats significantly decreased the expression of proapoptotic executioner caspase-3 enzyme [76]. Furthermore, Met has been reported to prevent the activation of caspase-3 when administered 24 h prior to DOX administration in a cell line of cardiomyocytes [77]. Alongside Met, previous investigations indicated that other drugs and natural products as Sitagliptin and Sophocarpine may mitigate DIC by inhibiting oxidative stress and apoptosis [76, 78].

As mitochondrial dynamics imbalance induced by DOX stimulated cardiomyocytes̕ mitochondrial structural abnormalities which are related to key cellular functions including ROS and Ca^2+^ signaling, energy metabolism, apoptosis [79]. Our findings suggest that ROS generation, calcium dysregulation triggered by DOX could be blocked by Met and support the hypothesis that Met could prevent DOX-induced apoptosis through protective effects on mitochondrial function**.**

In conclusion, the results of this study indicate that Met may attenuate DIC through antioxidant and anti-apoptotic actions while maintaining mitochondrial dynamics balance and calcium homeostasis. To our knowledge, this is the first study to explore the effect of Met on mitochondrial dynamic imbalance and calcium dysregulation in DIC in vivo. As a result, our findings pave the way for clinical trials to investigate Met as a potential chemoprotective medication in combination therapy with DOX to reduce cardiotoxicity.

## Data Availability

The data that support the findings of this study are available upon reasonable request.
